# Stereotypes of incompetence in schizophrenia among mental health professionals

**DOI:** 10.1192/j.eurpsy.2022.1588

**Published:** 2022-09-01

**Authors:** K.-M. Valery, A. Prouteau, T. Fournier, L. Violeau, S. Guionnet

**Affiliations:** University of Bordeaux, Psychology, Bordeaux, France

**Keywords:** schizophrénia, stigmatization, Incompetence stereotype, mental health professionals

## Abstract

**Introduction:**

Mental health professionals are one of the major sources of stigma for persons with schizophrenia and their families. The stereotype of incompetence is central in this stigmatization, whereas valuing skills is a fundamental aspect of mental health care and recovery.

**Objectives:**

The aim of this study is to identify the domains of competence stigmatized in schizophrenia by mental health professionals and the factors associated with this stigmatization.

**Methods:**

An online survey was conducted with a specific measure of the stereotype of incompetence and these associated factors. Participants were to be mental health professionals who work or have worked with persons with schizophrenia. These participants were recruited through professional social networks.

**Results:**

Responses of 164 participants were analyzed. The results reported four highly stigmatized skill domains: ability to relate well socially, ability to be effective in their work, ability to make decisions about their health, and ability to control their emotions. Intelligence was found to be less stigmatized than the other dimensions. Recovery beliefs, categorical beliefs, and perceived similarities were factors associated with the stereotype of incompetence.

**Conclusions:**

Responses of 164 participants were analyzed. The results reported four highly stigmatized skill domains: ability to relate well socially, ability to be effective in their work, ability to make decisions about their health, and ability to control their emotions. Intelligence was found to be less stigmatized than the other dimensions. Recovery beliefs, categorical beliefs, and perceived similarities were factors associated with the stereotype of incompetence.

**Disclosure:**

No significant relationships.

